# Beyond Dryness: Mapping the Psychological and Cognitive Burden in Sjögren’s Disease—A Narrative Review

**DOI:** 10.3390/jcm15082857

**Published:** 2026-04-09

**Authors:** Adriana Elena Neagu, Daniela Opriș-Belinski, Teodora Baciu, Sinziana Daia-Iliescu, Claudia Cobilinschi, Ioana Saulescu

**Affiliations:** 1 Department of Internal Medicine and Rheumatology, “Sfanta Maria” Clinical Hospital, 37-39 Ion Mihalache Blvd, 011172 Bucharest, Romania; teodora.baciu@rez.umfcd.ro (T.B.); sinziana.daia@umfcd.ro (S.D.-I.); claudia.cobilinschi@umfcd.ro (C.C.); ioana.saulescu@umfcd.ro (I.S.); 2 Department of Rheumatology and Internal Medicine, Carol Davila University of Medicine and Pharmacy, 050474 Bucharest, Romania

**Keywords:** Sjögren’s disease, dryness, neuropsychiatric manifestations, depression, anxiety, cognitive dysfunction, fatigue, mental health, brain fog, neuroimmune mechanisms, psychological distress, quality of life

## Abstract

**Background:** Sjögren’s disease (SjD) is a chronic systemic autoimmune disorder characterized by persistent exocrine gland inflammation, possible multi-organ involvement and a marked predominance of mid-life women. Beyond dryness and fatigue, patients report mood disturbances and cognitive complaints such as “brain fog”, which affect daily functioning and quality of life. **Objective:** To summarize and critically synthesize the literature on depression, anxiety, cognitive function, personality traits and quality of life assessment in adults with SjD and to highlight clinically relevant gaps. **Methods:** We performed a narrative review (PubMed, Cochrane, Embase through June 2025) of studies on psychological outcomes, cognitive function and quality of life in adults with SjD. **Results:** Depression and anxiety were frequently observed: depressive symptoms were present in roughly one-third to nearly half of patients, while anxiety symptoms were reported by about one-third. Cognitive impairment (affecting memory, attention and executive function) was also frequently described, often alongside severe fatigue and sleep disturbance. Overall, quality of life was reduced in SjD, driven mainly by fatigue and emotional distress rather than by classic disease activity. Neuroimmune mechanisms (e.g., chronic systemic inflammation and cytokine signalling such as IL-6 and TNF-α) may contribute to affective and cognitive symptoms. Overall, the evidence base remains largely cross-sectional and heterogeneous. **Conclusions:** Psychiatric symptoms and cognitive complaints represent a substantial and clinically relevant burden in SjD. Routine screening and multidisciplinary management that includes psychological assessment and support may improve well-being, adherence and quality of life.

## 1. Introduction

### 1.1. Background on Sjögren’s Disease

Sjögren’s disease (SjD) is a complex autoimmune systemic condition marked primarily by persistent inflammation of exocrine glands, especially those producing saliva and tears. As a result, patients frequently experience uncomfortable symptoms, notably dry mouth (xerostomia) and dry eyes (xerophthalmia). However, the effects of SjD often extend beyond these common manifestations, potentially involving several other organs and tissues, such as joints, lungs, kidneys and even the nervous system [1].

Epidemiological research has consistently highlighted that women are significantly more prone to developing SjD compared to men, with recent estimates suggesting a female-to-male ratio on average 9:1. The condition typically emerges during middle age, affecting individuals predominantly in their late 40s to mid-50s [1,2].

Population-based evidence indicates that SjD is relatively rare yet clinically and economically burdensome. In a French nationwide claims-based study (2011–2018), the prevalence of SjD ranged from 23 to 32 per 100,000 persons (≈0.023–0.032%), with women accounting for ~90% of cases, and incidence declined from 4.3 to 0.7 per 100,000 between 2012 and 2017. Annual healthcare costs increased over time, with median (mean) per-patient costs ranging from €2290 (€5780) to €4261 (€9710) [3]. Beyond sicca symptoms, extraglandular manifestations are reported in a substantial proportion of patients and the most serious long-term complication remains B-cell lymphoma, which occurs in ~5–10% of patients (7–15-fold increased risk vs. the general population), particularly in higher-risk subsets (e.g., persistent gland enlargement, purpura/vasculitis, cryoglobulinaemia, low complement) [4].

The development of Sjögren’s disease is shaped by a complex interplay of genetic predisposition, environmental triggers and immune dysregulation [5,6]. A defining immunological feature of SjD is the presence of anti-Ro/SSA and anti-La/SSB autoantibodies, which are detected in approximately 60–70% of patients, respectively 40–50% [5,7]. These autoantibodies are not only valuable for diagnostic purposes but have also been linked in several cohorts with earlier onset, more pronounced glandular dysfunction and a higher frequency of extraglandular features; however, effect sizes vary across studies [6,7]. In addition, seropositivity and/or higher titers of anti-Ro/SSA and anti-La/SSB antibodies have been associated in some cohorts with a greater likelihood of systemic disease expression, including neurological involvement and neuropsychiatric manifestations, although the available evidence remains heterogeneous and not all studies confirm a direct association. Although SjD may sometimes be perceived as relatively mild when compared with other autoimmune conditions [8], the persistent symptoms, particularly severe dryness of the eyes and mouth, as well as profound fatigue, can considerably disrupt everyday life and reduce overall quality of life [8,9]. In fact, severe fatigue alone affects around 70% of patients, significantly limiting their daily activities and underscoring the substantial personal burden posed by SjD [8].

In the past few years, the international medical community has progressively adopted the term “Sjögren’s disease” instead of “Sjögren’s syndrome.” This terminological change reflects a broader conceptual shift—from viewing the condition as a localized exocrinopathy to recognizing it as a systemic autoimmune disease with multisystem involvement and significant psychosocial burden. Notably, this evolution was not driven solely by academic consensus but also by strong advocacy from patient organizations, such as the Sjögren’s Foundation, who argued that the word “syndrome” minimizes the real-life impact of the illness. By adopting the term “disease,” clinicians and researchers acknowledge the complex interaction of dryness, fatigue, depression and cognitive dysfunction that profoundly shapes patients’ daily lives. This change in language therefore mirrors a deeper understanding of Sjögren’s as a chronic, multidimensional and disabling condition, extending well beyond glandular pathology [10].

### 1.2. Relevance of Psychological Status

Although traditionally defined through its somatic and immunological features, recent studies increasingly emphasize the psychological impact of Sjögren’s disease. Patients report persistent psychological symptoms, including chronic fatigue, anxiety, depression and cognitive impairment, which often show poor correlation with objective markers of disease activity or inflammation [1]. However, higher levels of fatigue have consistently been associated with greater depressive symptom scores [11]. As described for other immune mediated disorders (rheumatoid arthritis, ankylosing spondylitis), these psychological challenges not only occur frequently but significantly undermine quality of life and daily functioning, increasing the risk of disability [9,12]. Similar associations between psychological dimensions (including personality types), disease activity and quality of life have been demonstrated in rheumatoid arthritis and ankylosing spondylitis [12].

Furthermore, the chronic and often fluctuating nature of Sjögren’s disease, combined with frequently prolonged diagnostic delays, has been clearly associated with poorer health perceptions and reduced functional performance in patients who experience a longer time to diagnosis [13]. These findings underscore the broader negative impact of diagnostic delays, affecting both physical and psychological aspects of patients’ quality of life [13]. Additionally, symptoms such as chronic pain and fatigue often overlap with psychological conditions like depression and anxiety, complicating both clinical evaluation and treatment strategies [14,15,16]. This clinical complexity highlights the need for comprehensive, multidisciplinary approaches to patient management [10,17].

High-level evidence supports a major patient-reported burden in SjD. A meta-analysis of SF-36 questionnaire data (7 studies; 521 patients with SjD and 9916 healthy controls) found significantly lower scores across all domains, with role-physical limitations among the most affected [18]. Psychiatric symptoms are also more frequent: a systematic review/meta-analysis reported markedly higher depression prevalence in SjD (pooled OR 5.36; standardized mean difference 1.47 vs. controls) and a cross-sectional sample reports anxiety and depressive symptoms in approximately one-third of SjD patients (33.8% for anxiety and 36.9% for depression) [19,20]. Cognitive complaints (“brain fog”) are reported in Sjögren’s disease; using a validated symptom scale (BCSI), high cognitive symptom burden was observed in ~20% of patients (vs. 3% of controls) [21].

### 1.3. Objective

This review aimed to summarize and integrate the available evidence in adults with Sjögren’s disease regarding their psychological status, more precisely depression, anxiety, cognitive complaints/function and health-related quality of life, and to identify key knowledge gaps to inform clinical practice and future research.

## 2. Materials and Methods

We performed a narrative review of the literature addressing psychological outcomes, with a focus on depression, anxiety, cognitive complaints and objective cognitive performance, fatigue-related neuropsychological symptoms, personality-related factors and health-related quality of life (HRQoL). We prioritized high-level evidence (systematic reviews/meta-analyses, large observational cohorts and well-characterized clinical studies), complemented by smaller mechanistic, neuroimaging and instrument-validation studies when they contributed clinically meaningful context.

**Figure 1:**
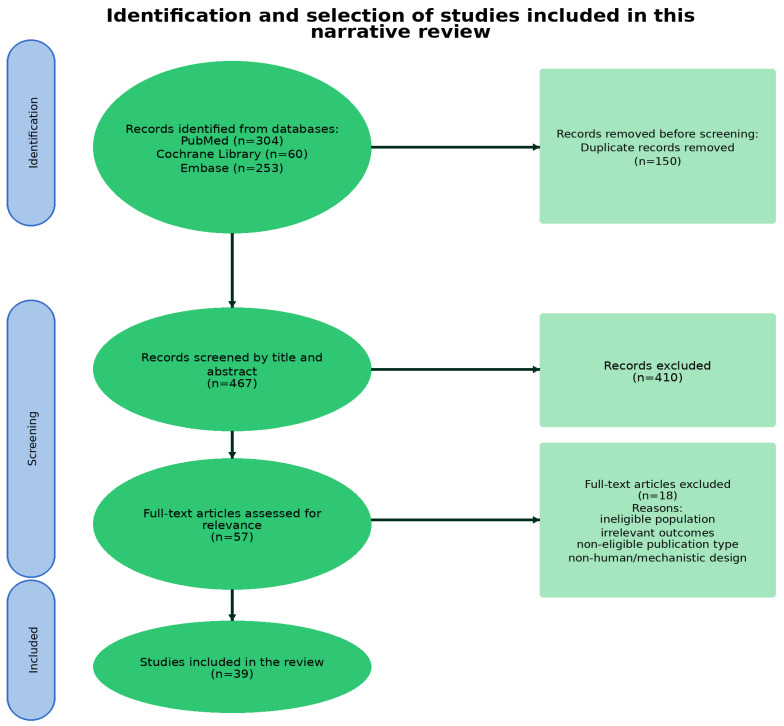
Flow diagram of literature search and study selection for this narrative review.

A focused literature search was conducted in PubMed, Embase and the Cochrane Library, followed by screening of titles, abstracts and full texts for relevance to the objectives of this narrative review. Although this review was narrative rather than systematic, the study selection process followed a structured multi-step process and is summarized in Figure 1 to improve transparency. After database searching and duplicate removal, titles and abstracts were screened for relevance. Full-text articles were assessed for eligibility when they addressed adult Sjögren’s disease populations and reported psychological, cognitive, fatigue-related, personality-related or HRQoL outcomes. Exclusion criteria comprised duplicate records, studies outside the scope of the review, pediatric or animal studies, editorials/letters/conference abstracts, publications without analyzable full text and articles lacking sufficient outcome data for qualitative synthesis. A detailed overview of the included studies, including study design, population, assessed outcomes, instruments and key findings, is provided in Supplementary Materials Table S1. When reported in the adopted papers, the original classification framework was preserved (including the 2002 AECG, 2012 SICCA/ACR and 2016 ACR/EULAR criteria) and no attempt was made to reclassify patients across studies, in order to maintain the integrity of the source definitions.

## 3. Results

The literature consistently indicates that Sjögren’s disease is associated with a substantial neuropsychological burden that extends beyond sicca symptoms. Across clinical cohorts and comparative studies, mood symptoms (particularly depression and anxiety), subjective cognitive complaints (“brain fog”) and impaired health-related quality of life (HRQoL) recur as prominent concerns. These outcomes are frequently intertwined with core symptom drivers such as fatigue, pain, sleep disturbance and perceived illness burden, which may amplify distress and shape patients’ day-to-day functioning. In the sections below, we synthesize the evidence thematically across key outcome domains and highlight clinically relevant patterns and sources of heterogeneity.

### 3.1. Psychological Comorbidities and Somatization in Sjögren’s Disease

Psychological comorbidities are among the most frequently reported and impactful non-exocrine manifestations in individuals with Sjögren’s disease. While SjD is traditionally characterized by its hallmark sicca symptoms, increasing evidence highlights that mood disorders, particularly depression and anxiety, are highly prevalent and significantly influence patient outcomes.

#### 3.1.1. Depression

Depression is a debilitating psychological condition in individuals living with Sjögren’s disease. Research shows that between 32 to 46% of patients with SjD experience clinically significant depressive symptoms [11,15,22,23,24], a rate markedly higher than that observed in the general population [19,20] and one study identifying 36.9% of SjD patients with depression compared to significantly lower rates in healthy controls [20].

What makes depression in SjD particularly challenging is that it does not consistently align with objective measures of cognitive performance or other disease parameters [22]. Instead, depression tends to correlate with more subjective and persistent symptoms, such as fatigue, chronic pain, dryness and cognitive complaints, which are often difficult to quantify or treat effectively [22,23]. These symptoms can deeply impact daily life [19], often going unrecognized in standard clinical assessments.

In a comparative study of 57 patients with SjD, 75 with SLE, and 199 with RA, Kotsis et al. [25] found that clinically significant depressive symptoms (PHQ-9 ≥ 10) were present in 24.6% of SjD, 29.3% of SLE, and 25.1% of RA patients. Importantly, in adjusted hierarchical regression models, illness perceptions and depressive symptoms were independently associated with physical HRQoL across all groups; in SjD, worries about disease consequences were a stronger correlate of physical HRQoL than pain [25].

Similarly, the meta-analysis and systematic review by Cui et al. [19] identified a pooled depression prevalence in SjD patients much higher than in controlled group (pooled OR 5.36; standardized mean difference 1.47 vs. controls) and highlighted the need of early recognition in order to minimize the possible impaired quality of life. The review also underscored that depression remains frequently underdiagnosed and undertreated in this population [19].

Adding a novel perspective, Zhu et al. [26] explored the role of the gut-brain-immune axis in SjD, showing that psychiatric symptoms, such as depression and anxiety, may be closely linked to changes in the gut microbiota. Their findings suggest a potential new target for better managing these symptoms, such as probiotics or other microbiota-modulating interventions, thus pointing toward a more integrative understanding of disease development and progression [26].

Overall, depression in SjD is a multifactorial phenomenon, with a focus on patients’ experience and how they interpret their illness. Addressing it requires more than immunosuppressive treatment: it calls for routine psychological evaluation, individualized mental health support and a broader approach to care [10,17].

#### 3.1.2. Anxiety

Anxiety is an impactful psychological comorbidity in Sjögren’s disease, often appearing alongside with depression and significantly diminishing quality of life [24]. While Cui et al. [19] focused primarily on depression in their meta-analysis, they also noted that anxiety frequently co-occurred across the included studies, underscoring its importance in this population [19].

In a large international cohort, Tarn et al. [27] used symptom-based stratification to identify a subgroup of SjD patients (approximately 25% of all patients) experiencing a high burden, with fatigue, pain, dryness, as well as elevated psychological distress, including anxiety and depression, regardless of systemic disease activity [27]. This suggests that anxiety in SjD, which can be found in one-quarter of patients, often stems from symptom experience rather than measurable disease processes.

Adding further nuance, in a cohort of SjD patients referred for cognitive complaints, Goulabchand et al. [14] reported clinically relevant trait anxiety in 48% alongside a high prevalence of sleep disturbances, indicating a two-way relationship across psychological and somatic domains [14]. On this note, a systematic review by Hackett et al. [28] found that sleep disturbances to be more prevalent in SjD than in comparator groups, reinforcing the clinical overlap between sleep disruption, fatigue and anxiety-related symptoms [28]. This reinforces the need to address sleep quality when managing anxiety and cognitive symptoms. Their findings support a multidimensional symptom model in which anxiety is woven into a broader web of patient experience.

On the note of the gut-brain-immune axis, in a cohort of 56 SjD-mediated dry eye patients, 30.4% met criteria for anxiety (HADS-A ≥ 8) and the anxious group had higher systemic disease activity (ESSDAI 8.0 ± 2.2 vs. 6.7 ± 1.8; *p* = 0.022) together with distinct gut microbiota differences (eg, lower Firmicutes/Bacteroidetes ratio, *p* = 0.027; Bacteroides expansion, *p* = 0.011; Actinobacteria depletion, *p* = 0.001) [29].

A valuable addition comes from Cui et al. [20] who conducted a cross-sectional study comparing 160 SjD patients to 170 healthy controls. They reported a prevalence of 33.8% for anxiety in SjD, significantly higher than in the control group. They also identified oral health issues and swallowing disorders as predictors of anxiety, while fatigue was a strong predictor of depression [20].

These insights reinforce the need for routine anxiety screening and interdisciplinary care strategies tailored to SjD patients [10,17], considering the intertwined nature of physical symptoms, mental health and overall disease perception. As an additional note, some authors have briefly discussed the possibility that psychological distress may occasionally manifest through physical symptoms, a process known as somatization [30,31]. While research on this concept in SjD is limited, acknowledging its potential may offer clinicians a broader perspective when evaluating persistent, unexplained symptoms in affected individuals.

### 3.2. Cognitive Impairment, Neuroimaging and Neurological Manifestations

#### 3.2.1. Cognitive Symptoms

Cognitive dysfunction is a frequently reported yet underappreciated manifestation in individuals with Sjögren’s disease. Many patients describe experiencing memory lapses, difficulty concentrating, mental slowing and even word-finding problems, symptoms often referred to collectively as “brain fog.” These issues, while subtle, can significantly interfere with daily activities and diminish quality of life.

First, in a recent narrative review, Salehi et al. [15] reported wide-ranging estimates for cognitive dysfunction in SjD, with prevalences spanning from 44% to 100%. In the same synthesis, depression prevalence was reported up to 32–46% and moderate-to-severe sleep disturbances were reported in 75% of patients. Based on these figures, the authors argue that psychological manifestations in SjD likely extend beyond stress tolerance alone and may reflect contributions from immune dysregulation, neuroinflammatory pathways and overall physical symptom burden [15].

Importantly, formal neuropsychological testing often confirms what patients report, showing deficits in general memory, executive skills and attention in those with SjD “brain fog” [14,21,22,23]. Using the Brief Cognitive Symptoms Inventory (BCSI), Segal et al. [21] found a higher prevalence of clinically relevant cognitive symptoms in Sjögren’s disease compared with controls (20% vs. 3%; 144 SjD vs. 35 controls), with BCSI scores showing moderate correlations with pain, depression, anxiety, fatigue and health-related quality of life [21]. In another study, same group compared 39 female SjD patients with 17 healthy controls and reported significantly higher depression, fatigue, pain and cognitive symptom scores in SjD, alongside inferior performance in psychomotor processing (*p* = 0.027) and verbal reasoning (*p* = 0.007); moreover, in regression analysis, depression and verbal memory independently predicted cognitive symptom burden, explaining 61% of its variance [22]. On the contrary, objective–subjective dissociation is also reported: in a matched case–control study (37 SjD vs. 37 controls), Epstein et al. [32] observed that patients reported greater fatigue, depressive feelings, autonomic symptoms and perceived memory lapses, but objective cognitive and memory measures showed only minimal differences between groups [32].

As for the severity of these cognitive symptoms, Seeliger et al. [33] evaluated 64 patients with neurological manifestations and found objective cognitive impairment in 55%, ranging from mild in 38% to severe in 17%. Another interesting fact, disease activity, assessed by ESSDAI, showed a significant association with the presence/severity of cognitive impairment, whereas disease duration was not associated [33].

A systematic review by Manzo et al. [34] (total of 6196 participants) corroborated the overall evidence that cognitive involvement can occur in Sjögren’s disease, most commonly described as subjective ‘brain fog’ or mild cognitive impairment. However, the reported frequency varied markedly across included studies depending on definitions and setting, ranging from 1.03% (4/415) in a cohort study to 78.8% (22/28) in a small case–control sample. The review also noted that cognitive symptoms may precede the diagnosis by ~2 years on average, suggesting that cognitive dysfunction can represent an early manifestation in some patients. Importantly, the authors argued that ‘brain fog’ is likely multifactorial, with pain, depression, sleep disturbances and medications among the most relevant determinants [34].

Further highlighting the complexity of this phenomenon, in a SjD cohort specifically referred for unexplained cognitive complaints, Goulabchand et al. [14] documented objective cognitive impairment in all patients (borderline impairment in 100%), with a pathological cognitive profile in 53% (hippocampal 37%, dysexecutive 22%, instrumental 16%) and these findings co-occurred with high prevalence of anxiety, depression and sleep disturbance. This suggests that psychological and physiological factors are deeply intertwined, with mental health playing a critical role in patients’ cognitive experience [14].

#### 3.2.2. Neuroimaging and “Neuro-Sjögren”

Advances in neuroimaging have begun to reveal biological correlates underlying cognitive impairment in SjD. In a resting-state fMRI case–control study, Hu et al. [35] evaluated 68 SjD patients and 69 healthy controls and found that SjD reduces static regional homogeneity in left orbital medial frontal gyrus, left caudate nucleus and right precuneus, areas crucial for attention, working memory and executive processing, while dynamic analyses additionally showed altered activity in frontal networks, including the left supplementary motor area (a region involved in action initiation and executive control). These imaging abnormalities correlated significantly with worse performance on neuropsychological tests, suggesting a tangible neurobiological basis underlying cognitive impairment in SjD [35].

Similarly, Blanc et al. [36] prospectively assessed 25 SjD patients (vs. 25 matched multiple sclerosis patients and 25 healthy controls) and identified cognitive disorders in 60%, with deficits most often involving processing speed, attention, memory and executive function. Importantly, white matter lesion burden on MRI correlated with cognitive impairment severity, supporting a structural substrate for cognitive dysfunction in SjD [36].

In this review, we use the term ‘Neuro-Sjögren’ to denote Sjögren’s disease with neurological involvement, a phenotype most often characterized by peripheral neuropathy, but also cranial nerve affection or central nervous system involvement [33,37,38]. In a Neuro-Sjögren cohort, Seeliger et al. [33] reported objective cognitive impairment in 55% of patients, spanning from mild (38%) to severe (17%) dysfunction, supporting clinically relevant CNS-associated cognitive involvement within neurologically enriched SjD phenotypes [33].

#### 3.2.3. Neurological Manifestations: From Subtle to Severe

Beyond cognitive dysfunction, SjD can present with a broad spectrum of neurological complications affecting the peripheral, central and autonomic nervous systems. In a recent systematic review, Fahad et al. [39] found that peripheral neuropathy was reported as the most frequent manifestation (up to 65% in one cohort, predominantly sensory), while CNS involvement was identified in 30% of cases in another cohort. More severe phenotypes were also described, including transverse myelitis (20%) and CNS vasculitis linked to severe neurological decline (15%). Autonomic dysfunction was reported in 50% of patients in a cohort study. Collectively, these data underline that although neurological involvement may be clinically heterogeneous and often cohort-dependent and it can substantially impact daily functioning and psychosocial well-being [39].

Blanc et al. [36] highlighted that dementia-like presentations, although uncommon in SjD overall, can occur in clinically selected cohorts: in their prospective SjD series, dementia was identified in 5/25 patients (20%), with cognitive decline potentially mimicking neurodegenerative disorders. These observations support the need for structured neuropsychological assessment and neurological work-up in SjD patients presenting with cognitive complaints, particularly when symptoms suggest progressive decline [36].

Notably, a population-based study that compared neuropsychiatric manifestation in 68 SLE patients with 72 SjD patients, found that several neuropsychiatric syndromes are similar as prevalence, including headache (87% vs. 78%), cognitive dysfunction (46% vs. 50%) and mood disorders (26% vs. 33%), but others differ, with cerebrovascular diseases being more common in SLE (12% vs. 3%) and polyneuropathies in SjD (18% vs. 56%) [40].

### 3.3. Personality Traits in Sjögren’s Disease

Personality traits were evaluated in a subset of the included studies using validated instruments grounded in the Five-Factor Model (FFM, “Big Five”), which captures five broad domains: neuroticism, extraversion, openness, agreeableness and conscientiousness. These traits are clinically relevant in chronic autoimmune disease because they can shape emotional regulation, coping strategies, illness perceptions and engagement with healthcare, potentially influencing patient-reported outcomes such as anxiety, depression and health-related quality of life. Accordingly, we synthesized the available evidence on personality profiles and their associations with psychological distress and quality of life in Sjögren’s disease.

Studies suggest that individuals with Sjögren’s disease may present with distinct personality characteristics. Milic et al. [41] compared 105 women with SjD with 52 rheumatoid arthritis patients and 54 healthy controls and found that both SjD and RA groups differed from controls by showing higher Neuroticism, lower Extraversion and Openness to experience, while Agreeableness and Conscientiousness did not differ. Depressive symptoms were similar to controls, but anxiety was higher. More precisely, high/very high Neuroticism (score ≥ 96) was present in 48.6% (54/105) of Sjögren’s disease patients versus 25.9% (14/54) of controls and clinically relevant anxiety in 26.7% (28/105) versus 5.6% (3/54) [41]

Additionally, Hyphantis et al. [30] explored psychological profiles (regarding defence styles and hostility dimensions) in relation to HRQoL in Sjögren’s disease compared with SLE and healthy controls. This comparison is particularly relevant because SLE is another systemic autoimmune disease frequently complicated by neuropsychiatric manifestations. The available data suggest that SjD and SLE share a substantial burden of psychological distress relative to controls, but that the SjD profile may not be fully superimposable, as personality-linked dimensions such as delusional guilt hostility and maladaptive defensive patterns remained clinically relevant within the SjD group [30]. In multivariable analyses within SjD patients, psychological distress was a consistent independent correlate of HRQoL, while personality-linked dimensions (notably delusional guilt hostility) retained independent associations with poorer physical HRQoL [30]. This profile suggests that personality-related factors may influence how SjD patients experience and express distress, with potential implications for patient–clinician communication and supportive care.

Overall, the available evidence although limited supports a personality-related signal in Sjögren’s disease characterized by greater negative emotionality (higher Neuroticism) and reduced positive social engagement (lower Extraversion), alongside defensive patterns (reduced humour and elevated guilt/hostility) that may contribute to impaired HRQoL beyond general psychological distress. Further longitudinal and methodologically consistent studies are needed to clarify directionality, disease specificity and the extent to which these traits inform targeted psychosocial or behavioural interventions.

### 3.4. Quality of Life and Fatigue

#### 3.4.1. Health-Related Quality of Life (HRQoL)

Patients with Sjögren’s disease often experience a substantial decline in health-related quality of life (HRQoL), with impairments that reported to be comparable to other autoimmune diseases such as rheumatoid arthritis or SLE and more pronounced than healthy controls [9,42]. These limitations are primarily driven by persistent, subjective symptoms like dryness, overwhelming fatigue and chronic pain, which underscores fatigue’s magnitude in SjD and its strong link to diminished HRQoL [8,24,42,43]. Moreover, a narrative review summarized additional factors potentially linked to impaired HRQoL in Sjögren’s disease, including pruritus, sleep disturbance, sexual dysfunction, pulmonary manifestations, psychological dysfunction and reduced physical functioning [42].

Pooled SF-36 evidence from a meta-analysis confirms substantially lower HRQoL in Sjögren’s disease versus healthy controls, particularly for role-physical functioning (≈35 points lower on the SF-36 0–100 scale) [18].

Further cross-sectional evidence supports broad HRQoL impairment. Liu et al. [24] assessed 304 patients with Sjögren’s disease (female cohort) using the SF-36 with comparison against population norms derived from a large Chinese general-population survey (*n* = 17,754), reporting reduced quality of life across all eight SF-36 domains (all *p* < 0.001). In multivariable analyses, symptom burden, particularly pain and fatigue, was associated with poorer HRQoL, alongside psychological symptoms; anxiety and depressive symptoms were common (42.4% and 40.78%, respectively) [24]

On the same note, in a large survey, Segal et al. [43] compared 277 patients with SjD versus 606 controls, assessing HRQoL and psychological burden. Patients reported significantly worse outcomes across all eight SF-36 domains (all *p* < 0.05) and higher symptom burden, including greater fatigue (FACIT-Fatigue 30.1 vs. 43.0) and a higher prevalence of depressive symptoms (CES-D ≥ 16: 37% vs. 12%), compared with controls [43].

Importantly, baseline data from a large therapeutic trial indicate that HRQoL impairment aligns more closely with patient-reported symptoms than with disease activity indices. Cornec et al. [44] assessed 120 patients with active Sjögren’s disease using the SF-36, symptom severity measures including ESSPRI and disease activity via ESSDAI; in multivariable analyses, symptom burden (ESSPRI, especially pain and ocular dryness intensity) showed the strongest independent associations with reduced HRQoL, whereas systemic activity (ESSDAI) was not independently associated [44].

An additional but often underrecognized contributor to impaired quality of life in women with Sjögren’s disease is sexual dysfunction, which may be related to genital dryness, dyspareunia and the psychological distress associated with intimate symptoms [42].

To better capture these unique patient perspectives, Lackner et al. [45] developed and validated a disease-specific HRQoL instrument (PSS-QoL) in 75 Sjögren’s disease patients. The 25-item instrument showed good internal consistency and high test–retest reliability, with convergent validity supported by correlations with ESSPRI and EQ-5D pain/discomfort [45].

#### 3.4.2. Fatigue and Psychological Impact

Fatigue is a major contributor to symptom burden in Sjögren’s disease, affecting a large majority of individuals; approximately 70% of patients report disabling fatigue [8], while abnormal fatigue (FSS ≥ 4) was observed in 67% in a cross-sectional cohort [11].

Real-world evidence indicates that fatigue is highly prevalent in Sjögren’s disease. In a large cross-sectional study, Gairy et al. [46] analysed physician-reported patient record forms (*n* = 1879) and patient questionnaires (*n* = 888) and stratified patients by symptom-severity clusters. Fatigue was reported by 75–89% of patients across the 5 clusters and patient-reported fatigue worsened with increasing disease severity (as reported by physicians), reflected by lower FACIT-Fatigue scores (mean 35.6 in the mild group vs. 28.5 in the moderate vs. 21.9 in the severe one) alongside lower patient-reported health status (EQ-5D) scores [46].

In a five-year prospective cohort, Haldorsen et al. [47] reported largely stable fatigue over time in Sjögren’s disease; at baseline, 70.7% of patients had high fatigue (FSS > 4; mean FSS 4.78) and multivariable models did not identify clear clinical or laboratory predictors of fatigue change (with only weak/isolated associations with measures such as anti-SSA or salivary flow) [47]. In contrast, a systematic review by Mardale et al. [48] emphasized that fatigue in SjD is linked with biological markers (e.g., rheumatoid factor, ESR, IgG) [48].

Fatigue in Sjögren’s disease appears closely intertwined with sleep disturbance. In a cohort of SjD patients referred for unexplained cognitive complaints, Goulabchand et al. [14] reported a high prevalence of sleep problems (insomnia 77%, excessive daytime sleepiness 55% and high risk for sleep apnea 45%) and clinically relevant trait anxiety in 48% [14]. Consistent with this, a systematic review reported that sleep disturbances were more common in SjD than in comparator groups; for instance, one included study found moderate-to-severe sleep disturbance in 75% of patients and polysomnography data suggested a higher prevalence of sleep apnea (64% vs. 28% in healthy controls). This reinforces the idea that fatigue should be understood not in isolation, but as part of a cluster of interrelated symptoms, including mood disturbance and cognitive dysfunction.

Also on this note, Mardale et al. [48] highlighted that fatigue was exacerbated by nighttime symptoms such as pain and nocturia, that can cause sleep disturbances, which then impaired concentration and coping during the day. Furthermore, fatigue was closely associated with cognitive impairment involving memory, attention and executive functioning issues [48].

Adding nuance to this picture, comparative studies suggest that the daily pattern of fatigue may differ from other autoimmune conditions. van Oers et al. [49] found that, unlike patients with systemic lupus erythematosus (SLE) or rheumatoid arthritis (RA), individuals with SjD did not experience a typical decline in fatigue after waking up (in only 10% of SjD vs. 58% of SLE and 47% of RA). In fact, for many, fatigue either remained unchanged or worsened shortly after waking up, a pattern that appeared unique for them [49]. Supporting these insights, Godaert et al. [50] demonstrated that both general and physical fatigue were significantly elevated in SjD and SLE patients compared to controls and for SjD varied similarly throughout the day with less decline in the morning, even when adjusting for depressive symptoms [50].

Taken together, these studies offer a consistent message: fatigue in SjD is not a secondary or trivial symptom, it is central to the lived experience of the disease, deeply interconnected with emotional well-being, sleep, cognition and day-to-day functioning. As such, its assessment and management should be a routine component of care for individuals with SjD.

### 3.5. Psychosocial Factors

Sjögren’s disease not only affects patients physically but also disrupts key aspects of psychosocial well-being. This section explores the roles of social support, coping mechanisms and illness perception in shaping psychological outcomes among individuals with SjD.

#### 3.5.1. Social Support and Coping Mechanisms

Social relationships play a vital role in helping individuals with SjD navigate the psychological challenges of chronic illness. It is known that strong perceived social support is associated with better emotional well-being and lower rates of depression. Yet, because hallmark symptoms like fatigue and pain are largely invisible, patients can sometimes feel misunderstood, invalidated or isolated, a reality that may contribute to social withdrawal and heightened emotional distress.

In a retrospective case–control study, Karaiskos et al. [51] compared 47 patients with Sjögren’s disease with 35 disease controls (lymphoma) and 120 healthy controls, focusing on psychosocial exposures prior to disease onset. The Sjögren’s group reported a higher burden of negative stressful life events, exemplified by loss of beloved persons reported in 17.1% vs. 2.3% in lymphoma controls and 2.7% in healthy controls. In the same analysis, Sjögren’s patients also reported lower perceived social support and a more maladaptive coping profile than both control groups [51]

Conversely, the use of passive coping mechanisms has been associated with higher levels of psychological distress. This is echoed in the comparative study by Bucourt et al., [31] which assessed 4 groups of patients with chronic rheumatic diseases (SjD, rheumatoid arthritis, spondyloarthritis, fibromyalgia) and showed that patients with SjD had lower use of adaptive coping strategies, including lower “distancing from pain” and lower “ignoring pain sensations”. Catastrophizing was highest in fibromyalgia, while SjD showed intermediate values similar to rheumatoid arthritis and spondyloarthritis. Consistent with psychological burden, 21.7% of SjD patients met criteria for a current major depressive episode, compared with 70.8% in fibromyalgia, 47.9% in rheumatoid arthritis and 23.4% in spondyloarthritis [31].

In addition, Hyphantis et al. noted in a comparative study including 40 SjD patients, 56 SLE and 80 controls, that maladaptive defence mechanisms, such as somatization and help-rejecting complaints, were more prevalent among SjD patients, alongside a significantly lower use of humour defence [30]. This further underscores the need for psychological support and education to encourage adaptive responses and emotional regulation.

#### 3.5.2. Illness Perception and Psychological Adaptation

How individuals perceive their illness can profoundly influence their emotional and behavioural responses. In a comparative study, Kotsis et al. [25] assessed 57 patients with Sjögren’s disease, 75 with SLE and 199 with RS using the Brief Illness Perception Questionnaire alongside measures of depressive symptoms and HRQoL. Clinically significant depressive symptoms (PHQ-9 ≥ 10) were present in 24.6% of Sjögren’s patients (vs. 29.3% in SLE and 25.1% in RA). Compared with the other disease groups, Sjögren’s patients reported poorer illness comprehensibility (less understanding of their disease) and greater symptom attribution to illness (higher “identity”). In hierarchical regression models, illness perceptions and depressive symptoms were independently associated with physical HRQoL across all three conditions; within Sjögren’s disease, perceived consequences/worries about illness showed a stronger association with physical HRQoL than pain [25].

Similarly, Segal et al. [16] evaluated 92 SjD patients and found that negative illness perceptions, particularly catastrophizing thoughts about pain, were linked to increased pain severity in both seropositive and seronegative patients with SjD [16]. Their research emphasized that catastrophizing and viewing symptoms as overwhelming can worsen both pain and psychological outcomes.

These insights underscore the importance of patient education and psychological support to help reframe maladaptive illness beliefs and encourage more constructive coping and health behaviours.

## 4. Discussions

### 4.1. Key Messages

This narrative review maps a consistent psychological and cognitive burden in Sjögren’s disease beyond sicca symptoms. Across the included evidence, depressive symptoms affect roughly one-third to nearly half of patients, anxiety symptoms occur in about one-third, cognitive complaints are frequently reported and HRQoL is substantially reduced, largely driven by fatigue and emotional distress rather than classic systemic activity measures. Overall, the literature supports a “symptom cluster” pattern (fatigue–pain–sleep disturbance–mood–cognition) that deserves routine recognition in clinical care.

### 4.2. Mechanistic Considerations

#### 4.2.1. Sex Hormones and Gender Differences

Sjögren’s disease predominantly affects women, with roughly a 9:1 female-to-male ratio [1,2]. This pronounced female bias likely stems from a combination of sex-linked immunological and psychosocial factors. Immunologically, sex hormones strongly shape immune responses: estrogen generally enhances immunity (leading to higher antibody production and prolonged survival of autoreactive T cells), whereas progesterone and testosterone tend to be immunosuppressive, dampening immune reactivity [52]. For example, estrogen signalling drives B-cell hyperactivity and autoantibody class switching while reducing central immune tolerance (for instance, by downregulating the Autoimmune Regulator gene, AIRE, in the thymus), thereby heightening female susceptibility to autoimmunity; in contrast, androgens promote tolerogenic pathways that help protect males from developing autoimmune disease [52]. Notably, in Sjögren’s disease, local androgen deficiency in salivary gland tissues has been hypothesized to contribute to disease pathogenesis, especially since disease onset often peaks around menopause when systemic estrogen levels decline [53].

From a psychological perspective, women exhibit distinct patterns of stress reactivity and affect regulation that could influence autoimmune risk. For instance, women mount a more robust hypothalamic–pituitary–adrenal (HPA) axis response to stress than men, secreting higher levels of corticotropin-releasing factor (CRF), adrenocorticotropic hormone (ACTH) and cortisol. They also experience slower HPA axis negative feedback, leading to more prolonged elevations of stress hormones [54]. This heightened stress reactivity, combined with pronounced sex differences in affective disorders, suggests a greater psychological vulnerability in women. Epidemiological studies indicate that women have roughly twice the prevalence of major depression [55] and anxiety disorders [56] compared to men. Chronic stress and mood disturbances can in fact disrupt immune regulation via neuroendocrine–immune pathways, potentially exacerbating autoimmune inflammation. Taken together, these immuno-hormonal and psychoneuroendocrine factors provide a plausible basis for the female bias observed in Sjögren’s disease. The approximately 9:1 sex ratio may result from an interplay between biologically driven immune modulation and sex-specific psychological stress responses.

#### 4.2.2. The ‘Sickness Behaviour’ Model

A mechanistic framework often invoked across immune-mediated conditions is the ‘sickness behaviour’ model, in which sustained peripheral inflammation is proposed to contribute to fatigue, anhedonia/low mood and cognitive slowing through neuroimmune signalling pathways (e.g., cytokine-mediated blood–brain barrier effects and glial activation) [57]. Evidence from other autoimmune diseases supports inflammation–neurobehaviour links: in rheumatoid arthritis, circulating cytokines (including IL-6 and TNF-α) have been associated with depressive symptom burden in clinical cohorts and improvements in mood-related outcomes have been summarized under targeted anti-cytokine therapies in the broader RA literature [58,59]. Preclinical lupus models also support a role for neuroinflammatory mechanisms, with microglial/astrocytic activation linked to depressive-like behaviour and cognitive dysfunction and IL-6 signalling implicated in these effects [60,61]. In Sjögren’s disease, the clinical phenotype summarized in this narrative review is compatible with this symptom cluster: severe fatigue is reported in a substantial proportion of patients and cognitive complaints (‘brain fog’) and depressive symptoms are also common across included studies. However, SjD-specific mechanistic evidence directly linking circulating cytokines or neuroinflammatory markers to these neuropsychological outcomes remains limited within the included clinical literature. Therefore, applying a ‘sickness behaviour’ framework to SjD should be regarded as hypothesis-generating and warrants dedicated SjD-focused studies.

### 4.3. Clinical Implications: Interventions and Management

Management of psychological symptoms in Sjögren’s disease requires a comprehensive approach that addresses both physical and mental health components. Despite the high prevalence of depression, anxiety, fatigue and cognitive dysfunction in this population, mental health support remains underutilized in standard rheumatologic care.

Antidepressants, including selective serotonin reuptake inhibitors (SSRIs) and serotonin-norepinephrine reuptake inhibitors (SNRIs) are frequently used for comorbid depression and anxiety in SjD, generally extrapolating from evidence in the wider population and other chronic autoimmune diseases. However, the specific efficacy of these medications in SjD has not been rigorously demonstrated, as most clinical trials either exclude SjD or do not analyze it separately [1,10,17]. British Society for Rheumatology (BSR) guidelines (2025) recommend following general principles for mood and anxiety disorders, highlighting the importance of routine screening for all SjD patients and, where indicated, initiation of standard antidepressant therapy. They also emphasize regular monitoring for side effects, especially given the potential for certain antidepressants (such as tricyclics) to worsen sicca symptoms due to their anticholinergic properties [10]. Furthermore, the Brazilian Society of Rheumatology recently noted that the use of psychotropic drugs (e.g., SSRIs, SNRIs) has no large evidence of their efficacy in SjD, those recommendations are based only on case reports and speculated from other chronic diseases [17].

Immunomodulatory treatments, such as hydroxychloroquine, corticosteroids and some biologic agents, are primarily prescribed to control systemic or glandular inflammation. While there have been reports that fatigue and mood disturbances may improve with hydroxychloroquine or steroids in some patients, most large trials have found these effects to be inconsistent or modest at best [7]. On this note, it’s further highlighted that there is no robust evidence that conventional or biologic therapies impact fatigue, depression or cognitive dysfunction in SjD [17,62,63].

Targeted therapies for neuropsychiatric symptoms, such as modafinil or psychostimulants for severe fatigue and cognitive dysfunction, have been trailed on a case-by-case basis but lack strong supporting evidence for routine use. Recent consensus guidance suggests reserving such agents for highly selected, refractory cases and always in conjunction with non-pharmacologic interventions [17,64].

Cognitive-behavioural therapy (CBT) and psychoeducational programs have shown promise in small pilot studies and feasibility trials for reducing fatigue and emotional distress in SjD. For example, a systematic review by Hackett et al. found that most studies were small and preliminary, but CBT and psychoeducation were generally well accepted by patients and associated with improvements in fatigue and emotional well-being [65].

Recently, a randomized controlled trial demonstrated that a program combining CBT, graded exercise and education (the “FESSONA” protocol) significantly improved fatigue, depressive symptoms, adaptive coping strategies and health-related quality of life compared to standard care [66].

The principal pharmacologic and non-pharmacologic approaches discussed in this review, together with their target domains and level of supporting evidence, are summarized in Table 1.

Mindfulness-based stress reduction and other psychoeducational interventions are also recommended in guidelines as safe and potentially useful, although direct evidence in SjD is sparse. The British Society for Rheumatology guideline (2025) and the Brazilian Society of Rheumatology (2025) both recommend as adjuvants to standard care, offering psychological therapies, CBT and group education, especially for patients with persistent fatigue or mood symptoms [10,17].

Support groups and peer-led interventions provide additional value, particularly by addressing the feelings of isolation, invalidation and uncertainty that are so commonly reported by SjD patients. Group-based psychoeducation, patient associations and online communities have all been linked to improved mood, illness acceptance and resilience [17,65].

Furthermore, given the complex and overlapping nature of the disease, multidisciplinary management, involving not only psychologists, rheumatologists, but also mental health professionals, can help tailor strategies to individual needs, enhancing treatment adherence and quality of life.

### 4.4. Gaps in the Literature and Future Directions

Despite increasing recognition of the psychological impact of Sjögren’s disease, significant gaps remain in the literature. These limitations hinder the development of targeted, evidence-based interventions to support the mental health and cognitive well-being of affected individuals.

#### 4.4.1. Limitations of Current Studies

Several limitations constrain inference. The evidence base is dominated by cross-sectional designs, heterogeneous outcome measures and varying thresholds for defining depression/anxiety, fatigue and cognitive impairment, limiting comparability across studies and precluding causal conclusions. Sample sizes vary widely and some cohorts are clinically selected, while men, racial/ethnic minorities and younger patients remain underrepresented. Moreover, as this is a narrative review, we did not perform a formal risk-of-bias appraisal; accordingly, findings should be interpreted as an integrative summary of the available literature rather than as definitive effect estimates. Importantly, key symptom-overlap confounders, particularly fibromyalgia, chronic pain syndromes, sleep disorders and primary mood disorders, were inconsistently assessed or adjusted for, limiting the extent to which the reported psychological and cognitive burden can be attributed specifically to Sjögren’s disease rather than to non-specific chronic-illness effects. Additionally, few studies adjusted for medication use or socioeconomic factors. This heterogeneity makes it difficult to draw firm conclusions about causality or to establish standardized treatment pathways.

#### 4.4.2. Suggestions for Future Research

To address these gaps, future research should prioritize:

- Longitudinal studies to clarify the progression of psychological symptoms and their relationship with disease activity over time.
- Larger, multicenter trials with well-defined inclusion criteria to improve generability and statistical power.
- Standardization of assessment tools, especially for fatigue, cognitive symptoms and quality of life to allow meaningful comparisons across studies.
- Neurobiological studies integrating neuroimaging and biomarker analyses to better understand CNS involvement and the pathophysiological basis of cognitive and emotional dysfunction.
- Intervention trials focused on psychological therapies (e.g., CBT, mindfulness) specifically adapted for the SjD population.
- Integrated care models assessing the effectiveness of multidisciplinary interventions on psychological and functional outcomes.

By addressing these priorities, future research can move the field toward more comprehensive, patient-centered care and evidence-based guidelines for the mental health management of SjD might be formulated.

Looking ahead, several emerging research avenues could help address the remaining gaps in understanding the neuropsychological burden of Sjögren’s disease. One such opportunity is the use of artificial intelligence and machine learning to integrate psychological, immunological and imaging data, potentially uncovering complex multi-system interactions that underlie cognitive and mood disturbances in Sjögren’s. Similarly, multi-omics approaches, particularly metabolomic profiling, may help identify novel biochemical signatures associated with neuropsychological symptoms and disease progression. Another promising frontier is the gut–immune–brain axis, with the possibility that gut microbiota dysregulation influences immune-mediated neuroinflammation in this disease. However, progress in these areas is currently constrained by the scarcity of dedicated studies and the absence of any validated neuropsychological biomarkers in Sjögren’s, underscoring the need for more comprehensive, interdisciplinary research to fill these critical gaps.

## 5. Conclusions

Sjögren’s disease extends far beyond sicca symptoms and fatigue, carrying a substantial psychological and cognitive burden and includes depression, anxiety, cognitive complaints, somatization and personality-related factors. Across the available evidence, these manifestations appear to reflect an interplay between immune/neuroinflammatory pathways, CNS involvement and psychosocial determinants such as illness perceptions and they are major drivers of impaired quality of life, often more than measurable systemic disease activity. However, psychological symptoms remain under-recognized and under-treated in routine care. Our findings support integrating structured screening and multidisciplinary, individualized management (including evidence-informed pharmacologic treatment for mood disorders and non-pharmacologic strategies such as cognitive-behavioural interventions, education and supportive care) alongside standard SjD therapy to improve well-being, functioning, adherence and outcomes that matter most to patients, while highlighting the need for more standardized, longitudinal, SjD-focused research to strengthen clinical translation.

## Figures and Tables

**Table 1:** Summary of pharmacologic and non-pharmacologic interventions for psychological and cognitive symptoms in Sjögren’s disease.

Type Of Intervention	Target Symptom/Domain	Level of Evidence
Antidepressants (SSRIs, SNRIs)	Depression, anxiety	Case reports and extrapolation from other diseases (no RCTs specific to SjD) [1,10,17]
Immunomodulatory therapy (hydroxychloroquine, corticosteroids, biologics)	Fatigue, mood disturbances (depression), cognitive dysfunction	Inconsistent results in trials; no robust evidence of benefit [7,17,62,63]
Psychostimulants (e.g., modafinil)	Severe fatigue, cognitive dysfunction	Case-by-case use; lack of strong evidence (reserved for refractory cases) [17,64]
Cognitive-behavioural therapy and psychoeducation	Fatigue, emotional distress (e.g., depression, anxiety)	Small pilot studies (preliminary evidence of improved fatigue and well-being) [65]
Combined CBT, graded exercise and education (“FESSONA” program)	Fatigue, depression, coping, quality of life	Randomized controlled trial (showed significant improvements vs. standard care) [66]
Mindfulness-based stress reduction	Stress, persistent fatigue or mood symptoms	Guideline-recommended adjunct (direct evidence in SjD is sparse) [10,17]
Support groups and peer-support interventions	Social isolation, mood, illness acceptance, resilience	Observational reports (linked to improved mood, acceptance and resilience) [17,65]

## Data Availability

No new datasets were generated or analyzed in this study. All information discussed in this review is derived from previously published studies cited in the reference list.

## Supplementary Materials

The following supporting information can be downloaded at: https://www.mdpi.com/article/10.3390/jcm15082857/s1, Table S1: Detailed overview of the included studies, including study design, population, assessed outcomes, instruments and key findings.

## Abbreviations

The following abbreviations are used in this manuscript:

ACTH	Adrenocorticotropic hormone
AIRE	Autoimmune Regulator gene
anti-La/SSB	Autoantibodies against La antigen
anti-Ro/SSA	Autoantibodies against Ro antigen
BCSI	Brief Cognitive Symptoms Inventory
BSR	British Society for Rheumatology
CBT	Cognitive-behavioural therapy
CES-D	Center for Epidemiologic Studies Depression Scale
CNS	Central Nervous System
CRF	Corticotropin-releasing factor
EQ-5D	EuroQol 5-Dimension questionnaire
ESR	Erythrocyte Sedimentation Rate
ESSDAI	EULAR Sjögren’s Syndrome Disease Activity Index
ESSPRI	EULAR Sjögren’s Syndrome Patient Reported Index
FACIT-Fatigue	Functional Assessment of Chronic Illness Therapy–Fatigue
FFM	Five-Factor Model
fMRI	Functional Magnetic Resonance Imaging
FSS	Fatigue Severity Scale
HADS	Hospital Anxiety and Depression Scale
HADS-A	Hospital Anxiety and Depression Scale—Anxiety subscale
HPA	Hypothalamic–pituitary–adrenal
HRQoL	Health-related quality of life
IgG	Immunoglobulin G
IL-6	Interleukin-6
MMSE	Mini-Mental State Examination
MRI	Magnetic Resonance Imaging
OR	odds ratio
RA	Rheumatoid arthritis
RCT	Randomised Controlled Trial
PHQ-9	Patient Health Questionnaire-9
PSS-QoL	Primary Sjögren’s Syndrome Quality of Life questionnaire
SjD	Sjögren’s disease
SF-36	36-Item Short Form Health Survey
SLE	Systemic Lupus Erythematosus
SNRIs	Serotonin-norepinephrine reuptake inhibitors
SSRIs	Selective serotonin reuptake inhibitors
TNF-α	Tumor necrosis factor-alpha

## References

[1] Negrini S., Emmi G., Greco M., Borro M., Sardanelli F., Murdaca G., Indiveri F., Puppo F. (2022). Sjögren’s Syndrome: A Systemic Autoimmune Disease. Clin. Exp. Med..

[2] Thurtle E., Grosjean A., Steenackers M., Strege K., Barcelos G., Goswami P. (2024). Epidemiology of Sjögren’s: A Systematic Literature Review. Rheumatol. Ther..

[3] Seror R., Chiche L., Beydon M., Desjeux G., Zhuo J., Vannier-Moreau V., Devauchelle-Pensec V. (2024). Estimated Prevalence, Incidence and Healthcare Costs of Sjögren’s Syndrome in France: A National Claims-Based Study. RMD Open.

[4] Nocturne G., Pontarini E., Bombardieri M., Mariette X. (2021). Lymphomas Complicating Primary Sjögren’s Syndrome: From Autoimmunity to Lymphoma. Rheumatology.

[5] Sandhya P., Kurien B., Danda D., Scofield R. (2017). Update on Pathogenesis of Sjogren’s Syndrome. Curr. Rheumatol. Rev..

[6] Baldini C., Chatzis L.G., Fulvio G., La Rocca G., Pontarini E., Bombardieri M. (2024). Pathogenesis of Sjögren’s Disease: One Year in Review 2024. Clin. Exp. Rheumatol..

[7] Mariette X., Criswell L.A. (2018). Primary Sjögren’s Syndrome. N. Engl. J. Med..

[8] Ng W.F., Bowman S.J. (2010). Primary Sjogren’s Syndrome: Too Dry and Too Tired. Rheumatology.

[9] Meijer J.M., Meiners P.M., Huddleston Slater J.J.R., Spijkervet F.K.L., Kallenberg C.G.M., Vissink A., Bootsma H. (2009). Health-Related Quality of Life, Employment and Disability in Patients with Sjögren’s Syndrome. Rheumatology.

[10] Price E.J., Benjamin S., Bombardieri M., Bowman S., Carty S., Ciurtin C., Crampton B., Dawson A., Fisher B.A., Giles I. (2025). British Society for Rheumatology Guideline on Management of Adult and Juvenile Onset Sjögren Disease. Rheumatology.

[11] Segal B., Thomas W., Rogers T., Leon J.M., Hughes P., Patel D., Patel K., Novitzke J., Rohrer M., Gopalakrishnan R. (2008). Prevalence, Severity, and Predictors of Fatigue in Subjects with Primary Sjögren’s Syndrome. Arthritis Care Res..

[12] Donisan T., Bojincă V.C., Dobrin M.A., Bălănescu D.V., Predețeanu D., Bojincă M., Berghea F., Opriș D., Groșeanu L., Borangiu A. (2017). The Relationship between Disease Activity, Quality of Life, and Personality Types in Rheumatoid Arthritis and Ankylosing Spondylitis Patients. Clin. Rheumatol..

[13] Meinecke A., Kreis K., Olson P., Hoeper J.R., Engelke F., Seeliger T., Skripuletz T., Ernst D., Hoeper K., Witte T. (2024). Impact of Time to Diagnosis in Patients with Primary Sjögren’s Syndrome: A Cross-Sectional Study. Clin. Exp. Rheumatol..

[14] Goulabchand R., Castille E., Navucet S., Etchecopar-Etchart D., Matos A., Maria A., Gutierrez L.A., Le Quellec A., de Champfleur N.M., Gabelle A. (2022). The Interplay between Cognition, Depression, Anxiety, and Sleep in Primary Sjogren’s Syndrome Patients. Sci. Rep..

[15] Salehi M., Zamiri A., Kim J., Texeira C., Shah K., Gunturu S. (2024). Exploring the Psychiatric Manifestations of Primary Sjögren’s Syndrome: A Narrative Review. Int. J. Rheumatol..

[16] Segal B., Pogatchnik B., Rhodus N., Sivils K.M., McElvain G., Solid C. (2014). Pain in Primary Sjögren’s Syndrome: The Role of Catastrophizing and Negative Illness Perceptions. Scand. J. Rheumatol..

[17] de Oliveira F.R., Appenzeller S., Pasoto S.G., Fernandes M.L.M.S., Lemos Lopes M.L., de Magalhães Souza Fialho S.C., Pinheiro A.C., dos Santos L.C., Valim V., Serrano E.V. (2025). Recommendations on Neurologic, Cognitive, and Psychiatric Manifestations in Patients with Sjögren’s Disease by the Brazilian Society of Rheumatology. Adv. Rheumatol..

[18] Zhang Q., Wang X., Chen H., Shen B. (2017). Sjögren’s Syndrome Is Associated with Negatively Variable Impacts on Domains of Health-Related Quality of Life: Evidence from Short Form 36 Questionnaire and a Meta-Analysis. Patient Prefer. Adherence.

[19] Cui Y., Li L., Yin R., Zhao Q., Chen S., Zhang Q., Shen B. (2018). Depression in Primary Sjögren’s Syndrome: A Systematic Review and Meta-Analysis. Psychol. Health Med..

[20] Cui Y., Xia L., Li L., Zhao Q., Chen S., Gu Z. (2018). Anxiety and Depression in Primary Sjögren’s Syndrome: A Cross-Sectional Study. BMC Psychiatry.

[21] Segal B.M., Rhodus N., Moser Sivils K.L., Solid C.A. (2014). Validation of the Brief Cognitive Symptoms Index in Sjögren Syndrome. J. Rheumatol..

[22] Segal B.M., Pogatchnik B., Holker E., Liu H., Sloan J., Rhodus N., Moser K.L. (2012). Primary Sjogren’s Syndrome: Cognitive Symptoms, Mood, and Cognitive Performance. Acta Neurol. Scand..

[23] Koçer B., Tezcan M.E., Batur H.Z., Haznedaroğlu Ş., Göker B., İrkeç C., Çetinkaya R. (2016). Cognition, Depression, Fatigue, and Quality of Life in Primary Sjögren’s Syndrome: Correlations. Brain Behav..

[24] Liu Z., Dong Z., Liang X., Liu J., Xuan L., Wang J., Zhang G., Hao W. (2017). Health-Related Quality of Life and Psychological Status of Women with Primary Sjögren’s Syndrome. Medicine.

[25] Kotsis K., Voulgari P.V., Tsifetaki N., Drosos A.A., Carvalho A.F., Hyphantis T. (2014). Illness Perceptions and Psychological Distress Associated with Physical Health-Related Quality of Life in Primary Sjögren’s Syndrome Compared to Systemic Lupus Erythematosus and Rheumatoid Arthritis. Rheumatol. Int..

[26] Zhu Y., Zhang K., Luo Z., Song Y., Wang X. (2025). Gut Microbiota on Anxiety and Depression in Primary Sjogren’s Syndrome: A Novel Insight. Heliyon.

[27] Tarn J.R., Howard-Tripp N., Lendrem D.W., Mariette X., Saraux A., Devauchelle-Pensec V., Seror R., Skelton A.J., James K., McMeekin P. (2019). Symptom-Based Stratification of Patients with Primary Sjögren’s Syndrome: Multi-Dimensional Characterisation of International Observational Cohorts and Reanalyses of Randomised Clinical Trials. Lancet Rheumatol..

[28] Hackett K.L., Gotts Z.M., Ellis J., Deary V., Rapley T., Ng W.-F., Newton J.L., Deane K.H.O. (2016). An Investigation into the Prevalence of Sleep Disturbances in Primary Sjögren’s Syndrome: A Systematic Review of the Literature. Rheumatology.

[29] Zhang Y., Gan M., He Y., Liu T., Xu M. (2023). Anxiety Disorders and Gut Dysbiosis in Primary Sjögren’s Syndrome-Mediated Dry Eye Patients. Int. J. Gen. Med..

[30] Hyphantis T., Mantis D., Voulgari P.V., Tsifetaki N., Drosos A.A. (2011). The Psychological Defensive Profile of Primary Sjögren’s Syndrome Patients and Its Relationship to Health-Related Quality of Life. Clin. Exp. Rheumatol..

[31] Bucourt E., Martaillé V., Goupille P., Joncker-Vannier I., Huttenberger B., Réveillère C., Mulleman D., Courtois A.R. (2021). A Comparative Study of Fibromyalgia, Rheumatoid Arthritis, Spondyloarthritis, and Sjögren’s Syndrome; Impact of the Disease on Quality of Life, Psychological Adjustment, and Use of Coping Strategies. Pain Med..

[32] Epstein L.C., Masse G., Harmatz J.S., Scott T.M., Papas A.S., Greenblatt D.J. (2014). Characterization of Cognitive Dysfunction in Sjögren’s Syndrome Patients. Clin. Rheumatol..

[33] Seeliger T., Jacobsen L., Hendel M., Bönig L., Kristian Prenzler N.K., Thiele T., Ernst D., Witte T., Stangel M., Kopp B. (2020). Cognitive Impairment in Patients with Neuro-Sjögren. Ann. Clin. Transl. Neurol..

[34] Manzo C., Martinez-Suarez E., Kechida M., Isetta M., Serra-Mestres J. (2019). Cognitive Function in Primary Sjögren’s Syndrome: A Systematic Review. Brain Sci..

[35] Hu C., Wang L., Huang J., Shang Y., Zhang X., Du X., Cao H., Han Z., Wei P. (2025). Regional Brain Function Study in Patients with Primary Sjögren’s Syndrome. Arthritis Res. Ther..

[36] Blanc F., Longato N., Jung B., Kleitz C., Di Bitonto L., Cretin B., Collongues N., Sordet C., Fleury M., Poindron V. (2013). Cognitive Dysfunction and Dementia in Primary Sjögren’s Syndrome. ISRN Neurol..

[37] Seeliger T., Prenzler N.K., Gingele S., Seeliger B., Körner S., Thiele T., Bönig L., Sühs K.-W., Witte T., Stangel M. (2019). Neuro-Sjögren: Peripheral Neuropathy with Limb Weakness in Sjögren’s Syndrome. Front. Immunol..

[38] Jaskólska M., Chylińska M., Masiak A., Siemiński M., Ziętkiewicz M., Czuszyńska Z., Smoleńska Ż., Zdrojewski Z. (2020). Neuro-Sjögren: Uncommon or Underestimated Problem?. Brain Behav..

[39] Fahad S., Khan A., Thapa P., Khan M.S., Jogiyat S., Moustafa W., Ririe A.K., Zahid R., Rajput J. (2025). Spectrum of Neurological Complications in Sjögren’s Syndrome: A Comprehensive Review. Cureus.

[40] Harboe E., Tjensvoll A.B., Maroni S., Gøransson L.G., Greve O.J., Beyer M.K., Herigstad A., Kvaløy J.T., Omdal R. (2009). Neuropsychiatric Syndromes in Patients with Systemic Lupus Erythematosus and Primary Sjögren Syndrome: A Comparative Population-Based Study. Ann. Rheum. Dis..

[41] Milic V., Grujic M., Barisic J., Marinkovic-Eric J., Duisin D., Cirkovic A., Damjanov N. (2019). Personality, Depression and Anxiety in Primary Sjogren’s Syndrome—Association with Sociodemographic Factors and Comorbidity. PLoS ONE.

[42] Miyamoto S.T., Valim V., Fisher B.A. (2021). Health-Related Quality of Life and Costs in Sjögren’s Syndrome. Rheumatology.

[43] Segal B., Bowman S.J., Fox P.C., Vivino F.B., Murukutla N., Brodscholl J., Ogale S., McLean L. (2009). Primary Sjögren’s Syndrome: Health Experiences and Predictors of Health Quality among Patients in the United States. Health Qual. Life Outcomes.

[44] Cornec D., Devauchelle-Pensec V., Mariette X., Jousse-Joulin S., Berthelot J.-M., Perdriger A., Puéchal X., Le Guern V., Sibilia J., Gottenberg J.-E. (2017). Severe Health-Related Quality of Life Impairment in Active Primary Sjögren’s Syndrome and Patient-Reported Outcomes: Data From a Large Therapeutic Trial. Arthritis Care Res..

[45] Lackner A., Stradner M.H., Hermann J., Unger J., Stamm T., Graninger W.B., Dejaco C. (2018). Assessing Health-Related Quality of Life in Primary Sjögren’s Syndrome-The PSS-QoL. Semin. Arthritis Rheum..

[46] Gairy K., Knight C., Anthony P., Hoskin B. (2021). Burden of Illness among Subgroups of Patients with Primary Sjögren’s Syndrome and Systemic Involvement. Rheumatology.

[47] Haldorsen K., Bjelland I., Bolstad A.I., Jonsson R., Brun J.G. (2011). A Five-Year Prospective Study of Fatigue in Primary Sjögren’s Syndrome. Arthritis Res. Ther..

[48] Mardale D.-A., Opriș-Belinski D., Bojincă V., Bojincă M., Mazilu D., Păsăran E., Nițăa C., Groșeanu L., Berghea F., Bălănescu A.-R. (2024). The Physical and Psychosocial Impact of Fatigue among Patients with Sjogren’s Syndrome: A Systematic Review. J. Clin. Med..

[49] van Oers M.L., Bossema E.R., Thoolen B.J., Hartkamp A., Dekkers J.C., Godaert G.L.R., Kruize A.A., Derksen R.H.W.M., Bijlsma J.W.J., Geenen R. (2010). Variability of Fatigue during the Day in Patients with Primary Sjögren’s Syndrome, Systemic Lupus Erythematosus, and Rheumatoid Arthritis. Clin. Exp. Rheumatol..

[50] Godaert G.L.R., Hartkamp A., Geenen R., Garssen A., Kruize A.A., Bijlsma J.W.J., Derksen R.H.W.M. (2002). Fatigue in Daily Life in Patients with Primary Sjögren’s Syndrome and Systemic Lupus Erythematosus. Ann. N. Y. Acad. Sci..

[51] Karaiskos D., Mavragani C.P., Makaroni S., Zinzaras E., Voulgarelis M., Rabavilas A., Moutsopoulos H.M. (2009). Stress, Coping Strategies and Social Support in Patients with Primary Sjögren’s Syndrome Prior to Disease Onset: A Retrospective Case-Control Study. Ann. Rheum. Dis..

[52] Fairweather D., Beetler D.J., McCabe E.J., Lieberman S.M. (2024). Mechanisms Underlying Sex Differences in Autoimmunity. J. Clin. Investig..

[53] Konttinen Y.T., Stegajev V., Al-Samadi A., Porola P., Hietanen J., Ainola M. (2015). Sjögren’s Syndome and Extragonadal Sex Steroid Formation: A Clue to a Better Disease Control?. J. Steroid Biochem. Mol. Biol..

[54] Kokras N., Hodes G.E., Bangasser D.A., Dalla C. (2019). Sex Differences in the Hypothalamic–Pituitary–Adrenal Axis: An Obstacle to Antidepressant Drug Development?. Br. J. Pharmacol..

[55] Salk R.H., Hyde J.S., Abramson L.Y. (2017). Gender Differences in Depression in Representative National Samples: Meta-Analyses of Diagnoses and Symptoms. Psychol. Bull..

[56] GBD 2019 Mental Disorders Collaborators (2022). Global, Regional, and National Burden of 12 Mental Disorders in 204 Countries and Territories, 1990–2019: A Systematic Analysis for the Global Burden of Disease Study 2019. Lancet Psychiatry.

[57] Korte S.M., Straub R.H. (2019). Fatigue in Inflammatory Rheumatic Disorders: Pathophysiological Mechanisms. Rheumatology.

[58] Mrđa J., Tadić-Latinović L., Božić Majstorović L., Mrđa V., Mirjanić-Azarić B., Ovčina I., Vranić S., Popović-Pejičić S. (2025). Association of TNF-α and IL-6 Concentrations with Depression in Patients with Rheumatoid Arthritis. Curr. Issues Mol. Biol..

[59] Siuchnińska H., Minarowska A., Wasilewska E. (2025). Beyond Joints: Neuropsychiatric Benefits of TNF-α and IL-6 Inhibitors in Rheumatoid Arthritis—Narrative Review. Int. J. Mol. Sci..

[60] Nomura A., Noto D., Murayama G., Chiba A., Miyake S. (2019). Unique Primed Status of Microglia under the Systemic Autoimmune Condition of Lupus-Prone Mice. Arthritis Res. Ther..

[61] Reynolds J., Huang M., Li Y., Meineck M., Moeckel T., Weinmann-Menke J., Mohan C., Schwarting A., Putterman C. (2024). Constitutive Knockout of Interleukin-6 Ameliorates Memory Deficits and Entorhinal Astrocytosis in the MRL/Lpr Mouse Model of Neuropsychiatric Lupus. J. Neuroinflammation.

[62] Maleki-Fischbach M., Kastsianok L., Koslow M., Chan E.D. (2024). Manifestations and Management of Sjögren’s Disease. Arthritis Res. Ther..

[63] Vitali C., Minniti A., Pignataro F., Maglione W., Del Papa N. (2021). Management of Sjögren’s Syndrome: Present Issues and Future Perspectives. Front. Med..

[64] Marinho A., Delgado Alves J., Fortuna J., Faria R., Almeida I., Alves G., Araújo Correia J., Campar A., Brandão M., Crespo J. (2023). Biological Therapy in Systemic Lupus Erythematosus, Antiphospholipid Syndrome, and Sjögren’s Syndrome: Evidence- and Practice-Based Guidance. Front. Immunol..

[65] Hackett K.L., Deane K.H.O., Strassheim V., Deary V., Rapley T., Newton J.L., Ng W.-F. (2015). A Systematic Review of Non-Pharmacological Interventions for Primary Sjögren’s Syndrome. Rheumatology.

[66] Zhou X., Chen J., Colas C., Hupin D., Killian M. (2025). FatiguE in Sjögren’s Syndrome: A Randomised Controlled Trial of COmbined Non-PhArmacological Therapeutic Strategies (FESSONA). BMJ Open Sport Exerc. Med..

