# Supplementary material for: Beyond Dryness: Mapping the Psychological and Cognitive Burden in Sjögren’s Disease—A Narrative Review
Source: J Clin Med. 2026 Apr 9;15(8):2857. doi: 10.3390/jcm15082857 (PMC13116287; doi:10.3390/jcm15082857)
Supplement: Supplementary file 1 [file jcm-15-02857-s001.zip › jcm-4230573-supplementary.pdf]

Supplementary Table S1. Characteristics of included studies.

Ref	Study (first author, year)	Study design	Population / sample size	Outcomes assessed	Instruments / measures	Key finding(s) (as reported)
[9]	Meijer et al., 2009	Comparative study (SD vs. controls)	235 primary (pSS) and secondary Sjogren (sSS)	HR-QOL, employment and disability	SF-36, an employment and disability questionnaire	All Sjogren patients scored lower on HR-QOL than controls, but sSS < pSS
[11]	Segal et al., 2008	Cross-sectional observational cohort	94 SD patients	Fatigue prevalence/severity and predictors (including relationships with pain, helplessness, depression)	VAS fatigue, Fatigue Severity Scale (FSS), Profile of Fatigue (ProF: somatic ProF-S and mental ProF-M)<br>Predictors: VAS pain; depression via CES-D	Abnormal fatigue (FSS ≥4): 67% of subjects.<br>Depression (CES-D ≥16): 32%.<br>Strongest predictors of fatigue (FSS and ProF-S): pain, helplessness, depression
[14]	Goulabchand et al., 2022	Prospective cohort	32 SD patients with memory complaints	Cognitive profiles, anxiety, depression, fatigue, sleep disorders and quality of life	Cognitive evaluation by neuropsychologists	All 32 patients showed at least borderline cognitive impairment and 17 (53%) exhibited a pathological cognitive profile: a hippocampal profile (37%), a dysexecutive profile (22%) and an instrumental profile (16%);<br>37% of patients were depressed, 48% exhibited a mild-to-severe anxiety trait.<br>Sleep disorders:

						excessive daytime sleepiness (55%), high risk for sleep apnea (45%), insomnia (77%)
[15]	Salehi et al., 2024	Narrative review (105 full-text articles)	NA	Depression & anxiety, Sleep disorders, Psychosis and Catatonia, Obsessive-Compulsive Disorder, Bipolar disorder, Cognitive dysfunction	NA	Depression prevalence 32–46%, cognitive dysfunction 44% -100%, moderate-to-severe sleep disturbances - 75% of patients
[16]	Segal et al., 2014	Cross-sectional observational study	92 SD patients	Pain severity and its psychological predictors (catastrophizing, illness perceptions, mood/anxiety/fatigue, pain anxiety)	Self-report questionnaire covering illness perceptions, pain catastrophizing, pain severity, mood, fatigue and pain anxiety	Catastrophizing predicted pain severity more strongly than age, fatigue, depression or anxiety in both seropositive and seronegative SD.<br>Pain catastrophizing + fibromyalgia status + serologic status + belief that illness would have severe consequences explained 55% of the variance in pain severity.
[18]	Zhang et al., 2017	Meta-analysis	7 studies; 521 SD patients vs 9,916 healthy controls	Health-related quality of life (HRQoL) across SF-36 domains	SF-36	Significantly lower SF-36 scores in SD across all 8 domains; greatest impairment in Role Physical (RP): MD −35.04 (95% CI −43.04 to −27.05)

[19]	Cui et al., 2018	Systematic review and meta-analysis	12 studies; total 1,917 patients SD vs. healthy controls	Depression (prevalence + severity/score vs controls)	PHQ-9; HADS; CES-D; Zung Depression Scale; BDI	Depression prevalence in SD patients much higher than in controlled group (pooled OR 5.36; standardized mean difference 1.47 vs controls, p<0.01)
[20]	Cui et al., 2018	Cross-sectional, single-center (hospital-based), July 2016–June 2017	160 SD vs. 170 healthy controls (age/sex matched)	Anxiety, depression; correlates with disease activity/symptoms; oral and swallowing domains	HADS-A/HADS-D; ESSDAI; ESSPRI; FSS; VAS pain; OSDI; OHIP-14; MDADI	Anxiety 33.8%; depression 36.9%. HADS-A 6.52±4.06 vs 3.02±2.78; HADS-D 6.71±4.32 vs 3.87±2.80 (SD vs controls)
[21]	Segal et al., 2014	Comparative study	144 SD vs. 35 controls	Cognitive function	The Brief Cognitive Symptoms Inventory (BCSI)	Higher cognitive symptoms than controls 20% vs. 3%.<br>BCSI scores correlated moderately with pain, depression, anxiety, fatigue and health quality
[22]	Segal et al., 2012	Cross-sectional case–control	39 SD vs. 17 controls	Subjective cognitive symptoms, depression, fatigue, pain, objective cognitive performance	Prof-M (Profile of Fatigue—Mental), CES-D, Fatigue Severity Scale (FSS), Short-Form McGill Pain Questionnaire; neuropsychological battery (HVLT-R, Stroop, Trail Making Test A/B, WCST, WAIS-III Digit Symbol, COWAT, BNT, WAIS-III Similarities, Benton JLO, WRAT-3 Reading)	Depression, cognitive symptoms, fatigue and pain scores were greater in the SD group; Patients with SD had inferior performance vs. controls in psychomotor processing and verbal reasoning; depression and verbal memory were independent predictors that accounted for 61%

						of the variance in cognitive symptoms
[23]	Koçer et al., 2016	Cross-sectional case–control	32 SD vs. 19 controls	Cognitive dysfunction (pattern/prevalence); depression; fatigue; QoL/health status; correlations between these domains	Neuropsych battery (MMSS/MMSE, Clock Drawing Test, COWAT, Stroop test patterns, PASAT, SDLT, AVLT, BNT, BJLOT, RCFT); depression (HDS + BDI); fatigue (FSS); QoL/health status (SF-36; EQ-5D1/EQ-5D2)	SD showed higher depression scores (40.6% vs 5.3%), higher fatigue, lower performance across multiple cognitive tests (all $p<0.05$) and a negative correlation between Clock Drawing and SF-36 Bodily Pain and General Health
[24]	Liu et al., 2017	Cross-sectional study	304 women with SD (China) vs. population norms from a general-population survey n=17,754	HRQoL; anxiety and depression; predictors of HRQoL	SF-36; Hospital Anxiety and Depression Scale (HADS)	SD patients had significantly lower SF-36 scores across all 8 domains vs controls (all $p<0.001$). Pain and fatigue were key negative predictors of HRQoL; anxiety and depressive symptoms were frequent (reported 42.4% and 40.78%, respectively).
[25]	Kotsis et al., 2014	Comparative cross-sectional study	57 SD vs. 75 SLE vs. 199 RA	Illness perceptions; depressive symptoms/psychological distress; physical HRQoL	PHQ-9; Symptom Check-List; Brief Illness Perception Questionnaire (B-IPQ); WHOQOL-BREF	Clinically significant depressive symptoms (PHQ-9 ≥10): 24.6% SD vs 29.3% SLE vs 25.1% RA<br>SD patients reported lower illness comprehensibility and

						higher symptom attribution (“identity”) vs SLE/RA.
[26]	Zhu et al., 2025	Narrative review	NA	Depression & anxiety in SD; gut microbiota dysbiosis and gut–brain axis pathways	NA	Gut microbiota may modulate CNS and behavior via bidirectional gut–brain mechanisms; dysbiosis is discussed as potentially contributing to SD progression and may relate to higher disease activity
[27]	Tarn et al., 2019	Multicentre observational cohort analysis + external validation cohorts + reanalysis of RCT datasets	Discovery cohort: UK Primary Sjögren's Syndrome Registry (UKPSSR) n=608 Validation cohorts: France (ASSESS) + Norway (Stavanger), total n=396	Symptom-based patient stratification (pain, fatigue, dryness, anxiety, depression) and comparison of clinical/biological profiles across symptom clusters	ESSPRI, HADS	Identified four symptom-based subgroups: Low Symptom Burden (LSB), High Symptom Burden (HSB), Dryness-Dominant with Fatigue (DDF), Pain-Dominant with Fatigue (PDF). Subgroups showed distinct clinical/biological profiles.
[28]	Hackett et al., 2016	Systematic review with narrative synthesis	8 full-text papers comprising 9 studies; total 350 SD vs. healthy	Sleep disturbances and related sleep outcomes (subjective and objective), including daytime somnolence, night awakenings, and	A mix of self-reported and objective measures, including polysomnography	Compared with controls, SD patients reported greater subjective sleep disturbances and greater daytime somnolence and demonstrated more night

			controls and/or disease controls (e.g., RA, SLE, OA)	obstructive sleep apnea (OSA)		awakenings and pre-existing OSA
[29]	Zhang et al., 2023	Observational, single-center, cross-sectional study	56 patients with SD-mediated dry eye	Anxiety disorder prevalence and correlates.<br>ESSDAI<br>Dry eye severity/ ocular discomfort; Gut microbiota composition	HADS-A;<br>STAI-S;<br>ESSDAI, ESSPRI;<br>OSDI, TBUT, Schirmer test;<br>Fecal microbiota	Anxiety prevalence: 30.4% (17/56);<br>Anxiety vs non-anxiety: higher ESSDAI, higher OSDI category, longer disease duration; gut microbiota: lower Firmicutes/Bacteroidetes ratio, Bacteroides expansion, Actinobacteria depletion
[30]	Hyphantis et al., 2011	Comparative cross-sectional study	40 SD vs 56 SLE vs 80 healthy controls	Defensive styles; hostility; psychological distress; HRQoL	DSQ; HDHQ; SCL-90-R; WHOQOL-BREF	Compared with controls, SD had lower HRQoL, less humour defence, higher help-rejecting complaints and higher delusional guilt hostility<br>SCL-90-R Somatization in SjD (1.65 ± 0.76) higher than in both SLE (0.91 ± 0.77; $p = 0.009$) and HC (0.93 ± 0.62; $p < 0.001$)
[31]	Bucourt et al., 2021	Multicenter cross-sectional comparative study	23 SD vs. 48 fibromyalgia vs. 47 RA vs. 47 SpA	Psychological distress/adjustment, pain and fatigue, coping strategies and psychiatric comorbidity (anxiety/depression disorders)	GHQ-28, FSS, FIQ, CSQ, MINI (DSM-IV axis I)	Adaptive coping lower in SD vs comparators:<br>CSQ distancing from pain: SD 4.26 ± 3.48 vs RA 7.47 ± 4.62 vs SpA 6.48 ± 4.17 vs FM 5.00 ± 3.40.

						CSQ ignoring pain sensations: SD 7.96 ± 4.97 vs RA 11.43 ± 5.09 vs SpA 11.13 ± 3.82 vs FM 9.15 ± 4.08. Catastrophizing: SD 6.78 ± 3.10, RA 6.34 ± 3.55, SpA 7.30 ± 4.10, FM 8.10 ± 4.06
[32]	Epstein et al., 2014	Matched case–control study	37 SD vs 37 controls	Neuropsychiatric profile; subjective cognitive complaints vs objective testing	SF-36 (HRQoL); subjective self-ratings and objective measures of cognition/psychomotor function/memory	subjective>objective mismatch: SD patients reported greater fatigue, impaired physical functioning, feeling depressed and autonomic symptomatology vs. controls, but the majority of objective testing showed minimal between-group differences
[33]	Seeliger et al., 2020	Observational, single-center cohort, cross-sectional	64 SD with Neuro-Sjögren	Prevalence and severity of cognitive impairment; association with disease activity (ESSDAI) and disease duration	Neuropsychological testing (TAP battery + CERAD-PLUS subtests); ESSDAI; disease duration	Cognitive impairment was present in 55% of Neuro-Sjögren patients, with mild impairment 38% and severe impairment 17%; ESSDAI showed a significant association with the presentation of cognitive impairment, while disease duration did not.

[34]	Manzo et al., 2019	Systematic review	18 studies + 3 case reports; total n=6,196 SD participants	Cognitive impairment (brain fog/MCI/dementia) and reported correlates (fatigue, mood, pain, sleep, etc.)	Heterogeneous across included studies (subjective cognitive symptoms and/or objective neuropsychological testing; some studies used neuroimaging)	Reported cognitive-impairment rates varied widely across included studies (e.g., 1.03% (4/415) in one cohort vs 78.8% (22/28) in a small case–control sample)
[35]	Hu et al., 2025	Cross-sectional, case–control study	68 SD vs. 69 controls	Regional brain function changes (static/dynamic fALFF/ReHo) and their association with objective cognitive performance	rs-fMRI (sfALFF/sReHo/dfALFF/dReHo); MMSE; NCT-A; DST; SAS/SDS (psychological covariates)	Compared with controls, SD showed reduced sReHo in left orbital medial frontal gyrus, left caudate nucleus and right precuneus, plus dynamic increases in dfALFF (left SMA) and dReHo (right dorsolateral superior frontal gyrus).<br>Imaging alterations related to objective testing: sReHo vs NCT-A ($p = 0.005$) and dReHo vs DST ($p = 0.007$)
[36]	Blanc et al., 2013	Prospective case–control study	25 SD patients vs. 25 matched multiple sclerosis patients vs. 25 controls	Cognitive disorders/dementia; cognitive-domain performance; white matter lesions and correlation with cognitive impairment severity	Brief Repeatable Battery for Neuropsychological Examination (French version); brain MRI with Wahlund classification	Cognitive disorders as processing speed, attention, memory and executive function were identified in 60% of SD patients and dementia in 5 patients. White matter lesion burden correlated with cognitive impairment severity

[37]	Seeliger et al., 2019	Cross-sectional study	184 patients with severe polyneuropathy associated with limb weakness -> 44 fulfilled ACR–EULAR criteria for SD	Characteristics of "Neuro-Sjögren, SD with neuropathy with limb weakness	Routine diagnostic work-up including sicca evaluation; anti-SSA(Ro) serology; minor salivary gland biopsy (focus score ≥1) in seronegative cases; electrophysiological studies (axonal/demyelinating patterns); EFNS CIDP criteria	The majority of patients (93%) were diagnosed with SD after neurological symptoms appeared; Limbs were symmetrically involved in 84% of patients (57% tetraparesis, 27% paraparesis)
[38]	Jaskólska et al., 2020	Observational cross-sectional study	50 SD patients	Prevalence and the phenotype of peripheral neuropathies in SD	Neurological and rheumatological examination; nerve conduction studies of 9 peripheral nerves	36 patients (72%) fulfilled the criteria for the diagnosis of neuropathy: carpal tunnel syndrome (54%) and axonal sensorimotor neuropathy (22%) were the most common. Neurological symptoms preceded the diagnosis of SD in 8 patients
[39]	Fahad et al., 2025	Systematic review	8 included studies (2010–2024); sample sizes ranged 40–150 SD patients	Neurological complications in SD	Heterogeneous across included studies	Across included cohorts: peripheral neuropathy-most frequent (up to 65% in one cohort), CNS involvement 30% in one cohort (including cognitive dysfunction), transverse myelitis (20%) and CNS vasculitis associated with severe neurological decline (15%). Autonomic dysfunction was reported

						in 50% in a cohort study
[40]	Harboe et al., 2009	Comparative population-based study	68 SLE vs 72 SD patients	Prevalence/pattern of the 19 neuropsychiatric syndromes using ACR definitions	Standardized examinations by internal medicine/neurology/neuropsychology; cerebral MRI and neurophysiological studies	Headache, cognitive dysfunction and mood disorders were similar in both (87% vs 78%, 46% vs 50%, 26% vs 33%, respectively), but cerebrovascular disease more prevalent in SLE (12% vs 3%) and neuropathies more common in SD (mononeuropathy 0% vs 8%; polyneuropathy 18% vs 56%)
[41]	Milic et al., 2019	Comparative cross-sectional study	105 women with SD vs 52 RA vs 54 healthy controls	Personality traits; anxiety; depression	NEO-PI-R; Zung SDS; Zung SAS	Compared with controls, SD showed higher Neuroticism ($p=0.007$) and lower Extraversion ($p=0.001$) and Openness ($p=0.013$). Depression similar to controls ($p>0.05$), whereas anxiety was higher $p<0.0001$)
[42]	Miyamoto et al., 2021	Narrative review	NA	HRQoL; costs and work disability; determinants of HRQoL; evidence for interventions	Narrative synthesis of literature (frequently using SF-36 and EQ-5D across included studies)	HRQoL is markedly reduced in SD and broadly comparable to other chronic diseases (e.g., RA, SLE, fibromyalgia). Impaired

						HRQoL is consistently linked to fatigue and pain and has also been associated with ocular/oral involvement, pruritus, sexual dysfunction, sleep disturbance, pulmonary involvement, psychological dysfunction and reduced physical function.
[43]	Segal et al., 2009	Cross-sectional study	277 SD vs. 606 controls	HRQoL and symptom burden; depressive symptoms; predictors of SF-36 domains	SF-36; FACIT-Fatigue; PROFAD-SSI; Modified Brief Pain Inventory (BPI); CES-D; Thinking scale	Compared with controls, SD patients had worse scores across all 8 SF-36 domains and higher symptom burden. Fatigue was greater (FACIT-F 30.1 vs 43.0) and depressive symptoms were more prevalent (CES-D ≥16: 37% vs 12%).
[44]	Cornec et al., 2017	Cross-sectional analysis of baseline data from a randomized controlled trial (TEARS)	120 active SD	HRQoL impairment and determinants; relationship between symptoms (ESSPRI), disease activity (ESSDAI) and HRQoL	SF-36; ESSPRI; ESSDAI (plus clinical/lab and dryness measures per trial protocol)	SF-36 indicated marked HRQoL impairment. In multivariable analyses, patient-reported symptoms (ESSPRI), particularly pain and ocular dryness intensity, showed the strongest independent associations with HRQoL; systemic

						activity (ESSDAI) was not independently associated.
[45]	Lackner et al., 2018	Instrument development and psychometric validation (cross-sectional; test–retest)	75 SD	Development/validation of a disease-specific HRQoL questionnaire (PSS-QoL)	PSS-QoL (25 items); ESSPRI; ESSDAI; EQ-5D	PSS-QoL showed good internal consistency (Cronbach's α=0.892), convergent validity (correlation with ESSPRI r=0.755; EQ-5D pain/discomfort r=0.531) and high reproducibility (ICC=0.958, 95% CI 0.926–0.981).
[46]	Gairy et al., 2021	Multinational, cross-sectional, real-world quantitative survey	All SD patients Physician surveys: 316 PRFs: 1,879 PSCs: 888 (47% din PRF)	Fatigue prevalence and patient-reported health status (FACIT-Fatigue; EQ-5D) across physician-assessed severity/clusters; work/activity impairment	FACIT-Fatigue; EQ-5D-3L; EQ-5D-VAS	Fatigue prevalence 75–89%; FACIT-F (mild/moderate/severe) 35.6 / 28.5 / 21.9; EQ-5D-3L 0.8 / 0.7 / 0.5; EQ-5D-VAS 70.3 / 59.2 / 46.3
[47]	Haldorsen et al., 2011	Prospective cohort (5-year follow-up)	141 SD baseline; 122 follow-up (87%)	Fatigue over time and associations with clinical/serologic variables	Fatigue Severity Scale (FSS); fatigue visual analog scale (VAS); FACIT-F; SF-36 Vitality	Fatigue remained largely stable: high fatigue prevalence 70.7% at baseline and 72.1% at follow-up. Overall fatigue change was not consistently predicted by clinical or laboratory parameters (only weak/isolated associations), with a

						small decline in SF-36 vitality (37.4→34.4)
[48]	Mardale et al., 2024	Systematic review	19 studies included total SD subjects across included studies = 1815	Fatigue, predictors/biomarkers, sleep disturbances related to fatigue, cognitive concerns, daily functioning and quality of life; management/therapy approaches	Fatigue Severity Scale (FSS), Profile of Fatigue (ProF), ESSPRI (fatigue domain), VAS fatigue, Multidimensional Fatigue Inventory (MFI)	Predictive factors for higher fatigue in SD included associations with rheumatoid factor, ESR and IgG levels; Sleep disturbances, particularly nighttime pain and nocturia, were determinants of persistent daytime fatigue; Cognitive impairment = deficits in global memory, executive function and attention
[49]	van Oers et al., 2010	Observational comparative study	53 SD vs. 27 SLE vs. 61 RA vs. 31 healthy controls	Diurnal variation of fatigue	Self-rated fatigue across the day (repeated assessments)	In contrast to SLE and RA (which showed a post-waking decrease), SD patients did not show a typical decline in fatigue after waking; fatigue remained unchanged or worsened shortly after waking.
[50]	Godaert et al., 2002	Observational comparative study	28 SD vs. 20 SLE vs. 30 controls	Multiple fatigue dimensions (general, physical, reduced activity, reduced motivation, mental fatigue), diurnal variation	Repeated daytime assessments of fatigue dimensions	Both general and physical fatigue were higher in SD and SLE than in controls; controls and SLE showed fatigue that first decreased then increased, whereas SD

						showed an opposite pattern in the first part of the day
[51]	Karaiskos et al., 2009	Retrospective case–control study	47 SD vs. 35 disease controls (lymphoma) vs. 120 healthy controls	Negative stressful life events (major life events) and daily hassles prior to disease onset; coping strategies; perceived social support	Questionnaires assessing major life events and daily hassles; coping strategies questionnaire; MOS Social Support Survey	Compared with lymphoma controls and healthy controls, SD patients reported more negative stressful life events prior to disease onset, like loss of beloved persons reported in 17.1% vs. 2.3% in lymphoma controls vs. 2.7% in healthy controls
